# Effects of co administration of gonadotropins and letrozole during the ovarian stimulation on IVF outcome for poor responders: a systematic review and meta-analysis of randomized controlled trials

**DOI:** 10.1186/s12884-025-08127-5

**Published:** 2025-10-08

**Authors:** Faezeh Zakerinasab, Mersad Khalili, Fatemeh AbdollahyBiroon, Qumars Behfar, Reza Parsaee, Mahdis Sadat Miri, Arina Ansari, Fariba Arbab Mojeni, Niloofar Deravi, Ramina Fazeli, Mobina Fathi

**Affiliations:** 1 Mashhad University of Medical Sciences, Mashhad, Iran; 2 Semnan University of Medical Sciences, Semnan, Iran; 3 University of Maryland, Baltimore, MD USA; 4 Department of Neurology, Faculty of Medicine, University Hospital Cologne, University of Cologne, Cologne, Germany; 5 Master Student in Molecular Genetics, Transplant Research Center, Shiraz University of Medical Sciences, Shiraz, Iran; 6 Faculty of Pharmacy and Pharmaceutical Science, Tehran Medical Science, Islamic Azad University, Tehran, Iran; 7 Student Research Committee, School of Medicine, North Khorasan University of Medical Sciences, Bojnurd, Iran; 8 Student Research Committee, School of Medicine, Mazandaran University of Medical Sciences, Sari, Iran; 9 School of Medicine, Shahid Beheshti University of Medical Sciences, Tehran, Iran; 10 Student Research Committee, School of Medicine, Alborz University of Medical Sciences, Alborz, Iran; 11 SBUMS, Arabi Ave, Daneshjoo Blvd, Velenjak, Tehran 19839-63113 Iran; 12 Iran University of Medical Sciences, Hemat Highway, next to Milad Tower, Tehran, 1449614535 Iran

**Keywords:** IVF, Letrozole, Gonadotropin, Systematic review, Meta-analysis

## Abstract

**Background:**

Letrozole utilizing, an aromatase inhibitor, has been examined to improve pregnancy rate in poor ovarian response women. Increasing the dose of gonadotropins and enhancement of follicles’ sensitivity to androgens, are the possible mechanism of letrozole. This meta-analysis aimed to evaluate the effect of co-administration of letrozole and gonadotropin on IVF/ICSI outcomes in poor ovarian response women.

**Method:**

Relevant randomized controlled trials were obtained through search in several databases including PubMed, Scopus, Clinicaltrials.gov, Google scholar, and Cochrane Library. The RCTs investigating the effect of letrozole + gonadotropin versus gonadotropin reporting pregnancy outcome were selected.

**Results:**

After analyzing 13 RCTs comprising 1692 patients, letrozole was associated with a reduction in the dose (SMD: -147.96 (-180.49, -115.42), *P* < 0.01) and duration (SMD: -2.82 (-4.21, -1.42), *P* < 0.01 of gonadotropin administration. There was no evidence of a difference between groups in the number of retrieved oocytes, clinical pregnancy rate, or live birth rate. A small reduction in the number of transferred embryos was observed (SMD: -0.26 (-0.49, -0.02), *P* = 0.03), though this finding should be interpreted with caution given heterogeneity across studies. Overall, no significant improvement in reproductive outcomes was demonstrated.

**Conclusions:**

Despite the lack of significant improvement in pregnancy and live birth rate in the letrozole group, the dose and duration of gonadotropins decreased. This has the potential to reduce the side effects of gonadotropins. However, further studies are necessary to confirm its safety and whether its use translates into clinically meaningful benefits.

## Introduction

In vitro fertilization (IVF) is a treatment option for subfertility that involves ovarian stimulation, oocyte retrieval, and embryo transfer. The success rate of IVF relies on the maturation of multiple follicles in response to gonadotrophin stimulation [1]. However, poor responders may have difficulty producing an adequate number of mature follicles or retrieving enough oocytes during the controlled ovarian stimulation (COS) phase. They typically have fewer embryos with decent quality for transfer or cryopreservation as well as increased cycle cancellation incidence [2].

To define the poor ovarian responder, there is ongoing controversy and heterogeneity in the research conducted thus far, due to the numerous factors that can potentially hinder a patient’s response to treatment, including underlying conditions and demographic profile [3]. However, there have been two prominent and frequently cited definitions introduced. The first and commonly accepted definition - the Bologna criteria - identifies a woman as a POR patient if she meets at least two of the following three criteria: (i) advanced maternal age (≥ 40 years) or any other POR risk factor; (ii) previous poor ovarian response (≤ 3 oocytes retrieved or previous cycle cancelled); and (iii) abnormal ovarian reserve test results (antral follicle count [AFC] < 5–7 follicles or Anti-Mullerian hormone [AMH] < 0.5–1.1 ng/ml). Additionally, if a patient experiences two episodes of POR after maximal stimulation, she can be labeled as such, even if the other criteria are not met [4]. Besides the strength of this definition, it fails to account for responder heterogeneity [5], overlooks young PORs, and does not consider oocyte quality. As a result, a more nuanced definition, called POSEIDON, has been developed to incorporate both quantitative and qualitative parameters of the patient, based on age, ovarian follicle reserve biomarkers, ovarian sensitivity to gonadotrophins stimulation, and the number of oocytes recruited during an IVF cycle [6].

Compensating for the inadequate response, the total dose of gonadotropins employed is also increased, leading to demanding costs without significant improvement [2]. Despite recent advances in IVF technology, low implantation potential and relatively high incidence of POR (5.6–35.1%) remain a challenge [7]. Evidence suggests, during ovarian stimulation for IVF, multiple follicular developments can lead to higher-than-normal levels of estradiol and progesterone in the late follicular phase. This leads to reduced support for the corpus luteum due to pituitary suppression caused by the excess sex steroids and failure of the procedure. As a result, luteal phase insufficiency is commonly observed after ovarian stimulation, which requires hormonal support to be provided externally [8, 9]. Several methods and treatments have been suggested to improve pregnancy success rates for patients with poor ovarian response (POR). These approaches include using different methods to impede follicular negative feedback, increasing the dosage of gonadotropins, addition of adjuvant treatment to conventional treatment, and implementing modified natural IVF cycles [10, 11]. however, there is insufficient evidence to support any of them. Androgens appear to play an essential role in ovarian physiology and follicular growth. Studies suggest that adding androgens to ovarian stimulation enhances the effects of FSH in follicular maturation and granulosa cell proliferation [12]. Given this, the use of androgens as a treatment option for PORs has been explored in recent literature. One of the commonly tested approaches is the use of an aromatase inhibitor. letrozole is a third-generation, reversible aromatase inhibitor that has been tested in individuals with poor responses to IVF treatments. As an aromatase inhibitor, it promotes the growth and maturation of follicles by blocking the intra-ovarian conversion of androgens into estrogen. As a result, negative feedback to estrogen levels is suppressed, leading to an increase in gonadotropins [13]. This effect is also further enhanced by the sensitizing effect of androgens on follicles to FSH [14]. While previous studies have reported on the subject, they take a general approach to evaluate all recommended strategies for IVF success in poor responders or include non-randomized studies, which may introduce bias into the findings. Out study aims to perform a meta-analysis that includes evidence generated from randomized controlled trials (RCTs) on the effects of co-administration of gonadotropins and letrozole during the ovarian stimulation on IVF outcome for poor responders.

## Method

This meta-analysis explores the effect of co administration of gonadotropins and letrozole during the ovarian stimulation on IVF outcome for poor responders. To ensure the study’s rigor and transparency, the research protocol has been registered on the Open Science Framework (https://osf.io/bv9zm), and a checklist was used for the search strategy, screening, and data selection. The researchers adhered to the PRISMA (Preferred Reporting Items for Systematic Reviews and Meta-analyses) guidelines [15].

### Literature search

A comprehensive literature search was conducted up to May 12, 2023 to identify relevant randomized controlled trials (RCTs) and systematic reviews. The following databases were searched: Cochrane Library, PubMed, SCOPUS, Google Scholar, and Clinical Trials. To generate subsets of relevant citations, a combination of Medical Subject Headings (MeSH) and text words were used. Two subsets were created for studies of poor ovarian response, one for the term ‘response’ and the other for ‘reserve’. For both subsets, the words ‘poor’, ‘low’, ‘slow’, ‘inadequate’, ‘suboptimal’, ‘decreased’, and ‘diminished’ were included. Another subset was created for studies of IVF, including the terms ‘in vitro fertilization’ and ‘IVF’ as well as the phrase ‘fertilization in vitro’. A fourth subset was created for studies of letrozole using the terms ‘letrozole’ and ‘aromatase inhibitor’. The subsets were combined using the ‘AND’ operator to generate a relevant subset of citations, and a publication type filter was applied to obtain only RCTs and systematic reviews. The search strategy was adjusted to meet the search engine specifications for each database. No language or date restrictions were applied. Additionally, the reference lists of included studies and relevant systematic reviews were screened to identify additional studies that could be included. Two independent reviewers conducted the search and screened all identified records according to inclusion criteria for eligibility. Any discrepancies between the reviewers were resolved through discussion and consensus.

### Criteria for selecting studies

This meta-analysis established inclusion and exclusion criteria before conducting a literature search. Only randomized controlled trials (RCTs) were considered for inclusion if they met the following criteria: inclusion of women with poor ovarian response (POR), at least one study group received exclusively the letrozole + gonadotrophin regimen without any other adjuvant intervention, the control group was given GnRH analogs and gonadotropins without any adjuvant agents, and mandatory reporting of clinical pregnancy outcomes. Any studies using adjuvant treatments as the only stimulation agents without gonadotropins were excluded from the analysis.

### Extraction of relevant data

To determine eligibility, the title and abstract of each study were carefully examined, with studies that did not clearly match the inclusion criteria being excluded. The full text of remaining studies was evaluated to determine which ones met the eligibility criteria to include in data extraction process. Next, the following information was selected for extraction in three sets: (1) patient-specific factors (i.e. what POR criteria are met, Age, BMI, and the duration of infertility), (2) Study Design (i.e. sample size of control and study groups, details of IVF protocol, dose and details of letrozole protocol), (3) Outcomes (i.e. pregnancy rate, live birth rate, number of retrieved oocytes, number of transferred embryos,, serum estradiol concentrations according to the time of interventions, total dosage of gonadotropins administered, and the duration of gonadotropin treatment). For studies with incomplete or unclear outcome reporting, attempts were made to contact the corresponding authors by email to request additional information.

### Study quality assessment

The quality of included literature was evaluated by two independent authors (AA, FZ), according to the Joanna Briggs Institute (JBI) critical appraisal checklist for RCTs (https://jbi.global/critical-appraisal-tools). The checklist consisted of 13 questions with responses to the questions including “yes,” “no,” “unclear,” and “not applicable”. Any inconsistencies in the evaluations were resolved by a third author, (MF). Additionally, the GRADE approach was utilized to evaluate the quality of each outcome.

### Statistical analysis

This meta-analysis was performed on the effects of co administration of gonadotropins and letrozole during the ovarian stimulation on IVF outcome for poor responders’ patients compared to a control group using Stata version 15 (Stata Corp, College Station, TX, USA). After the authors have extracted data, meta-analysis has been on the adequate data. We have gathered all information to be systematically reviewed and explained in the result section. Standardized mean difference (SMD) between the control and the patient’s group was used as the main unit of analysis for each variable. The cut-off values have been set by Cohen for the interpretation of medium, small, and large effect sizes (0.5, 0.2, and 0.8, respectively). Analyses have been performed employing the random effects model. The authors have assessed heterogeneity by I2 statistics and values larger than 50% were announced as moderate to high heterogeneity. We also have done meta-regression when we found enough studies to examine the relationship between pregnancy rate, and live birth rate as potential effect modifiers. The credibility of evidence was evaluated by GRADE profiler v.3.6 software (The Cochrane Collaboration, Oxford, UK) in accordance with GRADE recommendations. Publication bias has been visually inspected through funnel plots and also quantitatively investigated using Begg’s and Egger’s regression test.

## Result

### Study selection and included studies

**Fig. 1:**
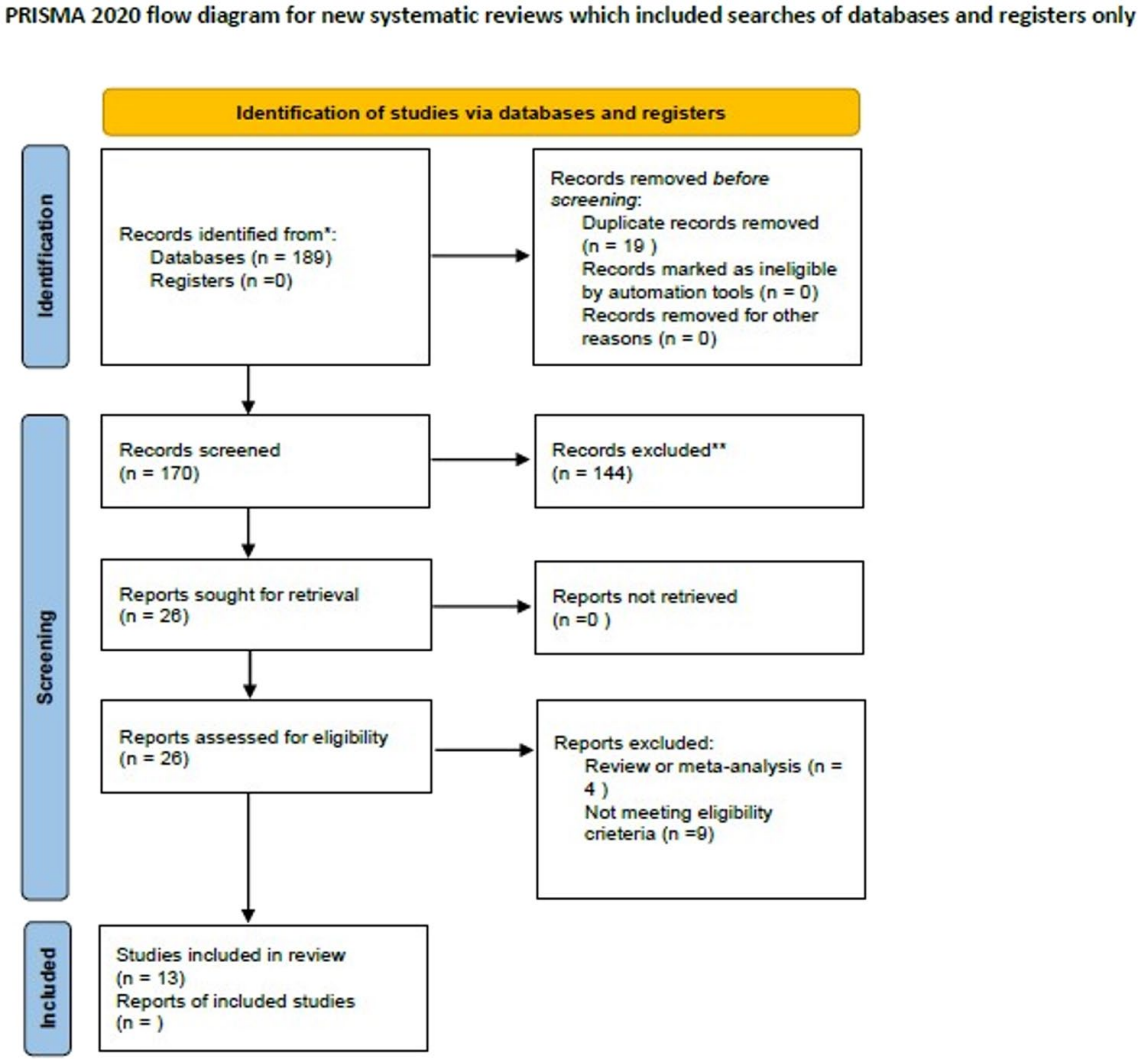
PRISMA flow diagram of the systematic review

Following a systematic search using MESH terms, a total of 189 studies were initially identified. Out of these, 19 duplicates were removed, leaving 170 studies for further assessment. After evaluating the abstracts of these articles, a total of 144 studies were excluded since they did not meet the eligibility criteria. The full text of the remaining 26 studies were retrieved, and 13 of them were excluded based on the reasons outlined in Fig. 1. Ultimately, only 13 studies matched the selection criteria and were included for meta-analysis. These eligible studies were published between 2008 and 2020 and involved a total of 1692 women who were identified as poor ovarian responders. Among these women, 754 received letrozole co-treatment during ovarian stimulation and were assigned to the intervention group, while the remaining 938 women received conventional COS and were placed in the control group. It is worth noting that the definition of poor ovarian response varied across studies due to some articles being published before the establishment of the Bologna criteria. Of the studies included, four came from Iran [16–19], three from China [20–22], two from Turkey [23, 24], and one each from Egypt [25], India [26], Denmark [27], and the USA [28]. All these articles sought to compare whether letrozole could enhance the IVF outcomes of patients diagnosed with poor ovarian response. While six studies used GnRH agonists, a total of nine RCTs employed GnRH antagonists. The letrozole co-treatment protocol slightly varied among the studies. For a more detailed description of the study characteristics, please refer to Table 1.

**Table 1:** Characteristics of the studies included

Publication, country of origin	Population (letrozol + control)	Inclusion criteria	intervention	Gonadotropin treatment	Stimulation protocol	Selected outcomes
Liu et al. [21] 2020, China	191 (97 + 94)	Bologna criteria	5 mg/day letrozol for 5 days (CD3-CD7)	L: 150 IU/day of r FSH (CD4 and CD6 and CD8) C: 300 IU/day of rFSH until trigger (CD start: NA)	Letrozol: Mild stimulation protocol C: controlled ovarian stimulation	1) Duration of gonadotropin treatment 2) Total doses of gonadotropin 3) No. of collected oocytes 4) No. of transferred embryos 5) Clinical pregnancy rate, Live birth rate,
Yu et al. [22] 2018, China	106 (52 + 54)	Age ≤ 42 years; AFC ≤ 8; AMH ≤ 1.5 ng/ml; FSH ≥ 15; BMI < 23; tubal factor or male factor	5 mg/day letrozol for 5 days (CD3-CD7)	L: 75 IU/day HMG since CD4 until trigger C: initial dose of 225–300 IU/day HMG based on body weight (then adjusted until trigger)	Letrozol: Mild stimulation protocol C: modified GnRH agonist protocol	1) Duration of gonadotropin treatment 2) Total doses of gonadotropin 3) No. of collected oocytes 4) No. of transferred embryos 5) Clinical pregnancy rate, Live birth rate,
Mohsen et al. [25] (2013), Egypt	60 (30 + 30)	Regular cycle; BMI < 30 AND one or more cycles with four or fewer oocytes and 300 FSH	5 mg/day letrozol for 5 days (CD2-CD6)	L: 150 IU/day hMG on CD7 untill trigger C: 300 IU/day hMG on CD3 untill trigger	L: Minimal stimulation protocol C: microdose flare up GnRH agonist protocol	1) Duration of gonadotropin treatment 2) Total doses of gonadotropin 3) No. of collected oocytes 4) No. of transferred embryos 5) Clinical pregnancy rate
Bastu et al. [23] (2016), Turkey	64 (33 + 31)	Bologna criteria, no metabolic or endocrine disorders; normal hormones; HSG/hysteroscopy	5 mg/day letrozol for 5 days (CD2/3-CD6/7)	75 IU FSH + 75 IU HMG; C: 150 IU FSH + 150 IU HMG	L: GnRh antagonist protocol C: GnRh antagonist protocol	Duration of gonadotropin treatment Total doses of gonadotropin No. of collected oocytes No. of transferred embryos Clinical pregnancy rate, Live birth rate,
Davar et al. [16] (2010), Iran	94 (45 + 49)	One or more failed IVF cycles with three or fewer follicles ≤ 16 mm and/or oestradiol on trigger day ≤ 500 pg/ml.	5 mg/day letrozol for 5 days (CD3-CD7)	300–450 IU FSH or HMG; C: 300–450 IU FSH	L: GnRh antagonist protocol C: MICRODOSE GNRH AGONIST FLARE PROTOCOL	Duration of gonadotropin treatment Total doses of gonadotropin No. of collected oocytes No. of transferred embryos Clinical pregnancy rate,
Goswami et al. [26] (2004), India	38 (13 + 25)	Age ≥ 35 years; one to three previous failed IVF owing to poor ovarian response	2.5 mg/day letrozol for 5 days (CD3-CD7)	L: 75 IU rFSH on CD3 and CD8 C: 300 IU/day FSH adjusted	L: gonadotropin C: GnRh agonist protocol	Total doses of gonadotropin No. of collected oocytes No. of transferred embryos Clinical pregnancy rate,
Ebrahimi et al. [17] (2017), Iran	70 (35 + 35)	Bologna criteria, Body mass index < 30 kg/m2, no other health problem and male infertility	2.5 mg/day letrozol for 5 days (CD3-CD7)	225 IU FSH adjusted; C: 225 IU FSH adjusted	L: GnRH-antagonist protocol C: GnRH-antagonist protocl	Duration of gonadotropin treatment Total doses of gonadotropin No. of collected oocytes No. of transferred embryos Clinical pregnancy rate,
Moini et al. [18] (2019), Iran	160 (80 + 80)	Bologna criteria, BMI < 30 kg/m2	5 mg/day letrozol for 5 days (CD2/3-CD6/7)	L: 150 IU/day of rFSH and 150 IU/day of HMG since CD1- trigger C: 150 IU/day of rFSH and 150 IU/day of HMG since CD1- trigger	L: GnRH-antagonist protocol C: GnRH-antagonist protocl	Duration of gonadotropin treatment Total doses of gonadotropin No. of collected oocytes No. of transferred embryos Clinical pregnancy rate,
Lee et al. [20] (2011), China	53 (26 + 27)	Fewer than four oocytes in a previous failed IVF cycles or AFC < 5.	2.5 mg/day letrozol for 5 days (CD2-CD6)	L: 225 IU/day hMG from CD7 untill trigger C: 225 IU/day hMG from CD3 untill trigger	L: CD7 225 IU HMG; C: CD3 225 IU HMG	Duration of gonadotropin treatment Total doses of gonadotropin No. of collected oocytes No. of transferred embryos Clinical pregnancy rate, Live birth rate,
Nabati et al. [19] (2015), Iran	123 (62 + 61)	Age > 40 years; previous cycle < 4; FSH 15–110 mIU/m and oestradiol < 1500 pg/m on CD3	2.5 mg/day letrozol for 5 days (CD3-CD7)	L: 450 IU/day FSH CD3 until trigger C: 450 IU/day FSH CD3 until trigger	L: GnRH-antagonist protocol; C: GnRh Agonist Microdose Flare Up	Duration of gonadotropin treatment Total doses of gonadotropin No. of collected oocytes No. of transferred embryos Clinical pregnancy rate,
Özmen et al. [24] (2009), Turkey	70 (35 + 35)	Previous cycle < 4 oocytes or previous cycle cancellation because of low oestradiol on CD6 or trigger day.	5 mg/day letrozol for 5 days (CD3-CD7)	L: 450 IU/day rFSH CD5 until trigger C: 450 IU/day rFSH CD3 until trigger	L: GnRh antagonist protocol C: GnRh antagonist protocol	Total doses of gonadotropin No. of collected oocytes No. of transferred embryos Clinical pregnancy rate,
Schoolcraft et al. [28] (2008), USA	514(179 + 335)	day 3 serum FSH > 10 mIU/mL, < 6 total antral follicles, prior cycle cancellation, prior poor response to COH, and age > 41	2.5 mg/day letrozol on CD3-CD7	L: 300 IU/day FSH and 150 IU/day HMG on CD3-trigger C:300 IU/day FSH and 150 IU/day HMG on CD5-trigger	L: GnRH antagonist protocol C: microdose GnRH agonist flare protocol	Duration of gonadotropin treatment Total doses of gonadotropin No. of collected oocytes No. of transferred embryos Clinical pregnancy,
Bülow et al. [27] (2021), Denmark	159 (80 + 79)	AMH 8–32 pmol/l; regular menstrual cycle; BMI < 35; not PCOS	5 mg/day letrozol on CD2/3-CD6/7	L: FSH 150 IU/day on CD2/3-trigger C: FSH 150 IU/day on CD2/3-trigger	L: GnRh antagonist protocol C: GnRh antagonist protocol	Duration of gonadotropin treatment Total doses of gonadotropin No. of collected oocytes No. of transferred embryos Clinical pregnancy rat

## Demographics

**Fig. 2:**
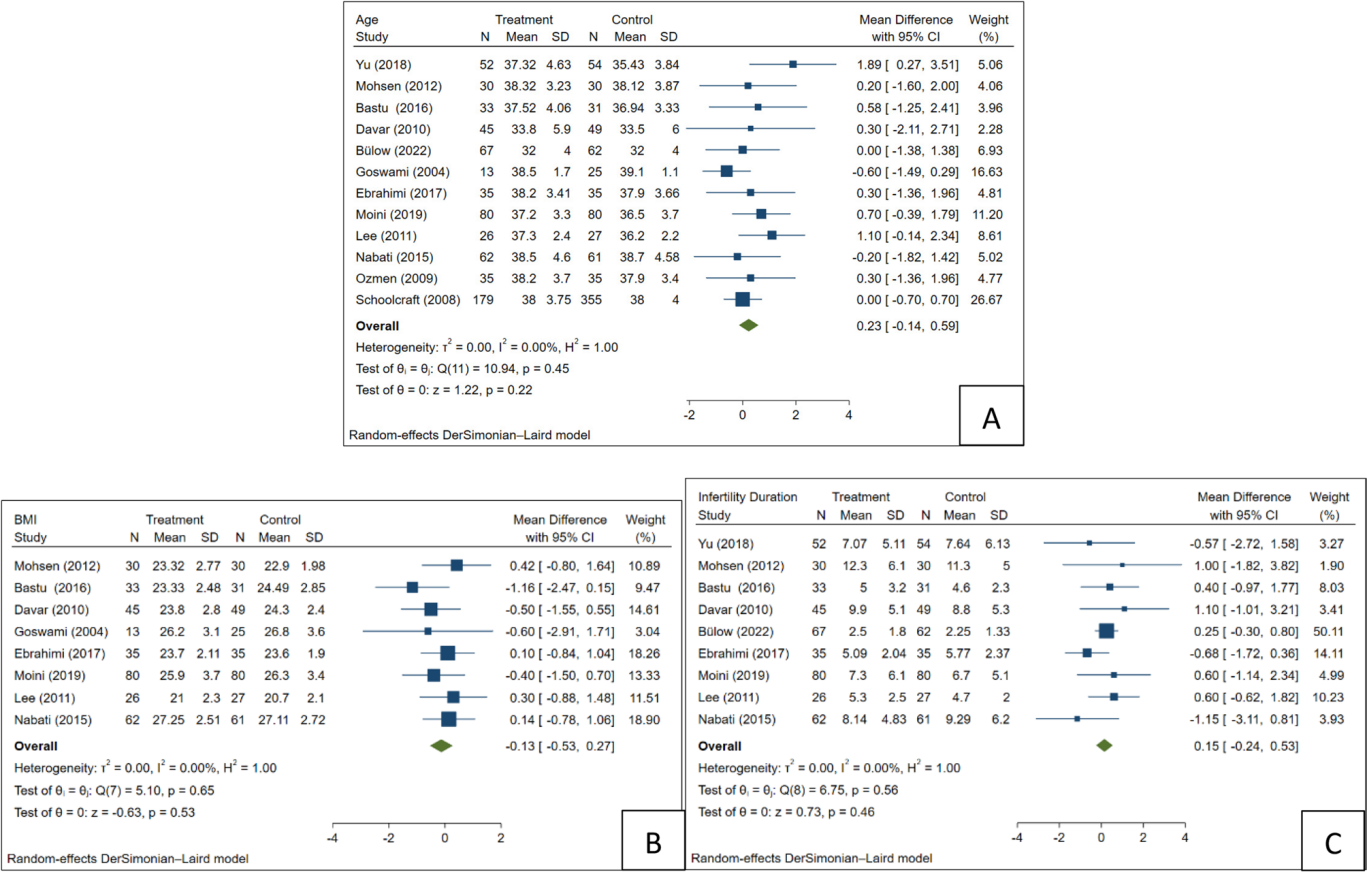
Forest plot for demographics; (**A**) Age, (**B**) BMI, (**C**) Duration of infertility

A total of 12, 8, and 9 studies reported the age [16–20, 22–28], body mass index (BMI) [16–20, 23, 25, 26], and duration of infertility [16–20, 22, 23, 25, 27] of the study participants, respectively. The analysis indicated that there was no significant difference (*P* > 0.05) concerning these demographic factors among studies, demonstrating no heterogeneity (I2: 0.0% for all three). Despite this, a random-effects model was still employed, as shown in Fig. 2; Table 2.

**Table 2:** The result s of meta-analysis for each variable

Variable	No. of studies	SMD [95%CI]	*P*-value
Age	12	0.23 [−0.14, 0.59]	0.22
BMI	8	−0.13 [−0.53, 0.27]	0.53
Infertility duration	9	0.15 [−0.24, 0.53]	0.46
No. of retrieved oocyte	12	−0.63 [−1.26, −0.01]	0.05
No. of transferred oocyte	11	−0.26 [−0.49, −0.02]	0.03
Total dose of gonadotropin	11	−147.96 [−180.49, −115.42]	< 0.01
Duration of gonadotropin stimulation	9	−2.82 [−4.21, −1.42]	< 0.01
		OR [95%CI]	
Pregnancy	12	−0.07 [−0.37, 0.23]	0.63
Live birth in total population	4	0.10 [−0.43, 0.62]	0.72
Live birth in pregnant population	4	0.18 [−0.69, 1.04]	0.69

### Number of retrieved oocytes

A total of 13 studies reported the number of retrieved oocytes. one of which [20] did not provide a standard deviation of data. Finally, 12 studies [16–19, 21–28], including 1639 patients (728 in the letrozole group and 911 in the control group), were eligible for the meta-analysis. There was no evidence of a difference in the number of retrieved oocytes between groups (SMD = 0.63, 95% CI= [−1.26, −0.01], *P* = 0.05). Subgroup analyses for both < 35 years and > 35 years women showed no statistically significant differences. Moreover, significant heterogeneity was observed among these studies (I2 = 86.87%); therefore, the random effects model was applied (Table 2).

### Number of transferred embryos

**Fig. 3:**
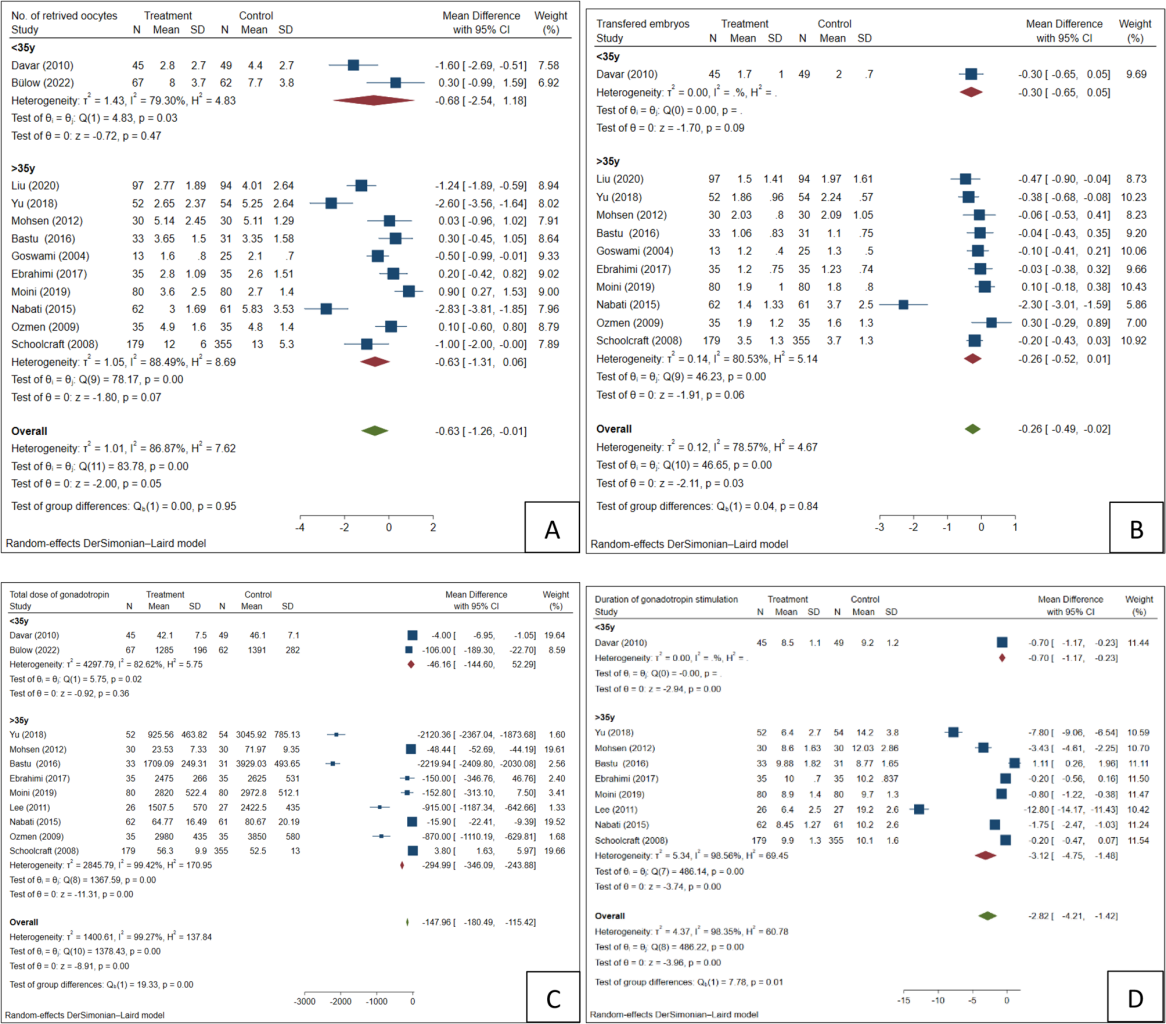
Forest plot for Secondary outcomes; (**A**) No. retrieved oocytes, (**B**) No. transferred embryos, (**C**) Total gonadotropin, (**D**) Duration of gonadotropin therapy

A total of 13 studies reported the number of transferred embryos, two of which [20, 27] did not provide standard deviation of data or lacked specific data required for a meta-analysis. The meta-analysis finally included 11 studies [16–19, 21–26, 28] for 1510 patients (661 in the letrozole group and 849 in the control group). The pooled analysis suggested fewer transferred embryos in the letrozole group compared to controls (SMD= −0.26, 95% CI= [−0.49, −0.02], *P* = 0.03). However, subgroup analyses by age (< 35 and > 35 years) did not show statistically significant differences, suggesting the overall effect may not be consistent across populations. The random effects model was performed because of the high heterogeneity among these studies (I2 = 78.57%), as shown in Fig. 3B; Table 2.

### Total dose of gonadotropin

A total of 13 studies reported a total dose of gonadotropin, two of which [21, 26] did not provide a standard deviation of data or lacked specific data required for a meta-analysis. The meta-analysis finally included 11 studies [16–20, 22–25, 27, 28] for 1501 patients (657 in the letrozole group and 844 in the control group). The result indicated a significant decrease in gonadotropin dosage with c0-administration of letrozole (SMD, −147.96, 95%CI= [−180.49, −115.42], *P* < 0.01). This reduction was particularly significant in women over 35 with SMD of −294.99 (95%CI= [−346,09, −243.88], *P* < 0.01]. However, for women under 35, the reduction was not significant (SMD=−46.16, 95%CI= [−144.60, 52.29]). The random effects model was used because of the high heterogeneity among these studies (I2 = 99.27%), as shown in Fig. 3C; Table 2.

### Duration of gonadotropin therapy

A total of 11 studies reported the duration of gonadotropin therapy, two of which [21, 27] did not provide a standard deviation of data or lacked specific data required for a meta-analysis. The meta-analysis finally included 9 studies [16–20, 22, 23, 25, 28] for 1264 patients (542 in the letrozole group and 722 in the control group). A significant decrease in the duration of stimulation was found in the letrozole group compared to the control group (SMD = −2.82, 95%CI= [−4.21, −1.42], *P* < 0.01). This effect was more pronounced for > 35 years old women (SMD= −3.12, 95%CI= [−4.75, −1.48], *P* < 0.01) versus < 35 years-old women (SMD= −0.70, 95%CI= [−1.17, −0.23], *P* < 0.01). The random effects model was performed because of the high heterogeneity among these studies (I2 = 98.35%), as shown in Fig. 3D; Table 2.

### Pregnancy

**Fig. 4:**
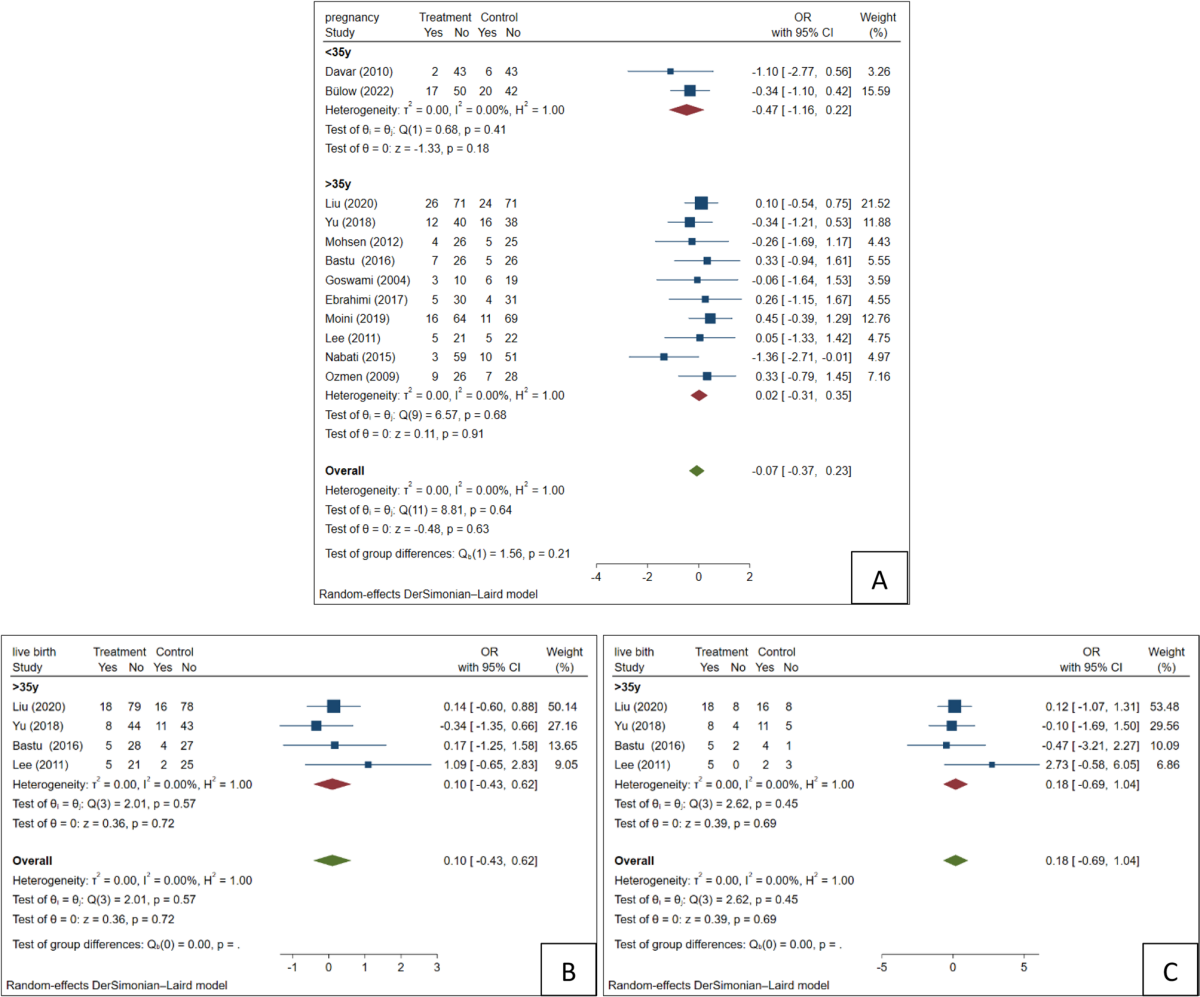
Forest plot of the (**A**) Pregnancy, (**B**) Live birth in total population, and (**C**) Live birth in pregnant population

A total of 12 studies [16–27] reported the number of pregnancies and underwent a meta-analysis. There was no statistically significant difference in pregnancy rate between the letrozole and control groups (OR= −0.07, 95%CI= [−0.37, 0.23], *P* = 0.63). The pregnancy result for women over 35 (OR= −0.47, 95%CI= [−1.16, 0.22], *P* = 0.18) aligned with the overall result. On the other hand, women under 35 exhibited a small positive effect with an OR of 0.02 (95%CI= [−0.31, 0.35], *P* = 0.91), although it remained insignificant (Fig. 4A).

### Live birth

Finally, 4 studies [20–23] reported data on live birth rate. We analyzed the chance of live births in the total and pregnant populations. There was no statistically significant difference in live birth rate between the groups (OR = 0.10, 95%CI= [−0.43, 0.62], *P* = 0.72). A similar non-significant pattern was observed among women who achieved pregnancy (Fig. 4B-C). No live birth rates were reported in any study participating women under 35, which made subgroup analysis impossible.

### Certainty of the evidence (GRADE)

The certainty of evidence was assessed using the GRADE approach for both primary and secondary outcomes (Table 3). Overall, the certainty ranged from moderate to very low. The evidence for pregnancy and live birth rates was rated very low, mainly because of imprecision, considerable heterogeneity, and the small number of trials reporting live birth. The outcomes related to the number of retrieved oocytes and transferred embryos were graded as low certainty, reflecting inconsistencies between studies and methodological limitations. By contrast, the findings on reduced gonadotropin dose and shorter stimulation duration were supported by moderate-certainty evidence, as the effect was consistent across trials despite differences in protocols.

**Table 3:** Certainty of the evidence (GRADE)

Outcome	No. of participants (studies)	Effect estimate	Certainty of evidence (GRADE)	Comments
Pregnancy rate	12 RCTs (1,159 women)	OR = −0.07 (95% CI: −0.37 to 0.23)	⬤◯◯◯ Very low	High heterogeneity; imprecision; inconsistent definitions of POR
Live birth rate	4 RCTs (414 women)	OR = 0.10 (95% CI: −0.43 to 0.62)	⬤◯◯◯ Very low	Few studies; wide confidence intervals; indirectness
No. of retrieved oocytes	12 RCTs (1,639 women)	SMD = −0.63 (95% CI: −1.26 to −0.01)	⬤⬤◯◯ Low	High heterogeneity; borderline significance
No. of transferred embryos	11 RCTs (1,510 women)	SMD = −0.26 (95% CI: −0.49 to −0.02)	⬤⬤◯◯ Low	Consistent direction but high heterogeneity
Total gonadotropin dose	11 RCTs (1501 women)	SMD = −147.96 (95% CI: −180.49 to −115.42)	⬤⬤⬤◯ Moderate	Consistent effect but heterogeneity in protocols
Duration of stimulation	9 RCTs (1,264 women)	SMD = −2.82 (95% CI: −4.21 to −1.42)	⬤⬤⬤◯ Moderate	Robust reduction but heterogeneous regimens

### Sensitivity and bias analysis

**Fig. 5:**
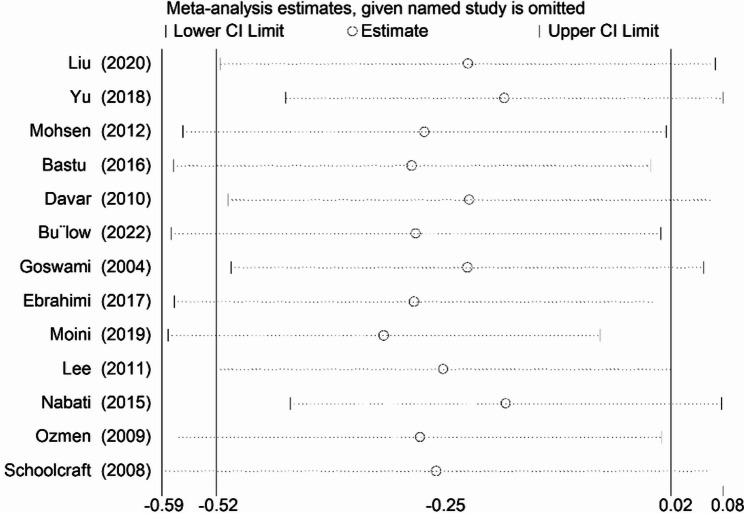
Leave-one-out sensitivity analysis plot

After conducting a sensitivity analysis, it was discovered that neither individual studies nor any clusters of studies that shared characteristics had any significant impact on the SMD and its corresponding CI (Fig. 5). These findings suggest that the overall results are robust. To assess the existence of publication bias, both Egger’s regression test and Begg’s test, as well as funnel plot analysis, were performed. Both Egger’s regression test and Begg’s test failed to reveal any evidence of publication bias (with *P* > 0.1), and the funnel plot analysis resulted in a symmetric plot for the number of retrieved oocytes (not shown). As a result, there was no indication of publication bias.

## Discussion

Letrozole, an aromatase inhibitor, can lower estrogen levels and increase androgen concentration within the ovary. This alteration can lead to improved follicular growth, enhanced ovarian response to stimulation protocols, and development of the follicular phase. Letrozole is commonly used as an adjuvant medication in infertility treatment alongside gonadotropin agonists or antagonists during IVF [29, 30]. Also, there is no evidence of teratogenicity for letrozole in terms of safety, and its rapid elimination from the body also reduces the likelihood of such effects.

Using higher doses of gonadotropins reduces the duration of stimulation but increases the total consumption of these hormones. However, increasing the dose of gonadotropins does not improve outcomes. Prolonged exposure to high doses of gonadotropins may sensitize FSH receptors and have a negative impact on oocyte and embryo quality [23].

The addition of letrozole to gonadotropins can decrease the need for high doses of gonadotropins and the duration of stimulation. It has a similar effect to receiving high doses of gonadotropins, ultimately leading to a reduction in the required dose of gonadotropins and cost savings [18, 23, 24, 26, 27, 31, 32].

This meta-analysis aimed to investigate the impact of co-administering gonadotropin and letrozole on the success of IVF by analyzing 13 RCTs with 1692 participants. The findings of this meta-analysis revealed no statistically significant difference in the clinical pregnancy rate and live birth rate between the case and control groups. However, letrozole successfully reduced the total dose and duration of gonadotropin administration along with a slight reduction in the number of transferred embryos.

Our findings are in line with several previous studies. Requena et al. [33] conducted a systematic review and meta-analysis of letrozole plus gonadotropins versus gonadotropins alone in IVF, including both randomized and non-randomized studies, and reported no significant difference in pregnancy rates per patient. Similarly, Bonardi et al. [34] found that the addition of letrozole to COS did not negatively affect mature oocyte yield or other efficacy outcomes, while it significantly reduced peak estradiol levels and gonadotropin use. These observations are consistent with our results, showing no change in reproductive outcomes but improved stimulation efficiency. However, their study population included women with hormone-sensitive cancers, which makes direct comparison with our infertility-focused cohort less straightforward.

In ovulation induction, the outcomes appear different. Baradwan et al. [35] reported that sequential letrozole/gonadotropin therapy improved ovulation and clinical pregnancy rates compared to letrozole alone in women with PCOS, also increasing endometrial thickness and mature follicle count. Likewise, Liu et al. [36] showed that letrozole monotherapy was superior to clomiphene citrate for ovulation, clinical pregnancy, and live birth rates in PCOS patients. These results likely reflect the different clinical contexts: in ovulation induction, the goal is to restore ovulatory function, where letrozole’s endocrine effects may play a stronger role, while in IVF, the natural ovulation process is bypassed, reducing the impact of these mechanisms [37].

Patient subgroups may also contribute to the heterogeneity across studies. Shapira et al. [38] showed in a self-controlled trial of poor and suboptimal responders that extended letrozole co-treatment during stimulation increased the number of retrieved oocytes, mature oocytes, 2PN embryos, and top-quality embryos, with a clinical pregnancy rate of 31.5%. Similarly, Bülow et al. [39], in a large meta-analysis of 31 studies, found that poor responders had a 7% absolute increase in live birth rate with letrozole co-treatment, without compromising oocyte yield, and with reduced gonadotropin use. In contrast, normal responders showed a higher oocyte yield but no improvement in live birth or clinical pregnancy rates, while evidence for high responders remained inconclusive.

Across the included trials, the findings were mixed but overall aligned with our results. Some studies reported higher ovarian response or oocyte yield with letrozole [18, 30], while others found better pregnancy or implantation outcomes with protocols that excluded letrozole [19, 28], possibly due to differences in oocyte or embryo quality and endometrial receptivity. Several trials reported no significant differences in reproductive outcomes, despite variations in hormonal changes or stimulation efficiency [17, 20, 40].

None of these individual trials contributed substantial weight to our meta-analysis, and sensitivity analyses confirmed that excluding any single study did not alter the overall direction of effect. Taken together, these findings suggest that while patient characteristics and protocol design may shape isolated outcomes, the overall evidence does not support a consistent improvement in clinical pregnancy or live birth rates with letrozole co-treatment in IVF.

The differences between individual trial findings and our pooled estimates are likely explained by heterogeneity in study design, patient populations, and intervention protocols rather than a uniform biological effect of letrozole. For instance, studies focused on poor responders often showed improved oocyte yield and embryo quality [38], whereas trials in normal responders or mixed groups generally did not find reproductive benefit [33, 34]. Variability in letrozole administration (from short 5-day courses to full-length co-treatment), differences in luteal support strategies (antagonist vs. progesterone-only), as well as protocol-related factors such as trigger methods, embryo transfer timing, and fresh versus frozen cycles, may also contribute to the inconsistent results.

Our meta-analysis showed that co-administration of letrozole significantly reduced gonadotropin dose and stimulation duration. Biologically, this can be explained by aromatase inhibition lowering estrogen synthesis, which increases endogenous FSH secretion and enhances follicular recruitment [41]. Clinically, these reductions may lower treatment costs, decrease patient burden, and potentially reduce the risk of OHSS; one analysis estimated savings of about USD 6,000 per clinical pregnancy [42]. However, the lack of a significant improvement in clinical pregnancy and live birth rates suggests that these gains in stimulation efficiency are not necessarily accompanied by improvements in oocyte competence or endometrial receptivity.

The high heterogeneity observed in our pooled analyses highlights the impact of differences in study design and patient populations on the overall effect estimate. While some observational studies have suggested comparable live birth rates with fewer complications when letrozole is used, the current evidence remains insufficient to confirm a consistent reproductive benefit across all IVF groups.

This meta-analysis has several strengths. By including only randomized controlled trials directly comparing gonadotropin plus letrozole with standard stimulation in women with poor ovarian response, the analysis was based on high-quality evidence. Adherence to a pre-registered protocol and PRISMA guidelines ensured transparency, while strict eligibility criteria minimized confounding from other adjuvants. The inclusion of diverse populations improved generalizability, and the evaluation of both stimulation parameters and reproductive outcomes provided a comprehensive overview. Subgroup and sensitivity analyses, together with assessment of publication bias, further strengthened the conclusions.

However, some limitations should be noted. Considerable heterogeneity persisted across studies in stimulation protocols, letrozole regimens, definitions of poor ovarian response, and laboratory practices. Although no language restrictions were applied, all included trials were published in English or Chinese, raising the possibility of selection bias. Incomplete reporting of statistical data limited inclusion of some trials, and reporting of key outcomes such as live birth was restricted to a small number of studies, preventing age-stratified analyses. Potential confounders, including ovarian reserve markers and lab conditions, were also inconsistently reported.

## Conclusion

In summary, based on the findings of this meta-analysis, there were no statistically significant differences in the clinical pregnancy rate and live birth rate when co-administering gonadotropin and letrozole in IVF. Future studies should focus on optimizing the dosage and duration of letrozole administration, considering factors such as patient characteristics, hormonal profiles, and ovarian response. Additionally, further investigation into the impact of letrozole on oocyte and embryo quality, endometrial receptivity, and the use of growth hormone in conjunction with letrozole could provide valuable insights for improving IVF outcomes.

## Data Availability

No datasets were generated or analysed during the current study.
